# Targeting Signaling Pathways in Epithelial Ovarian Cancer

**DOI:** 10.3390/ijms14059536

**Published:** 2013-05-02

**Authors:** Elisabeth Smolle, Valentin Taucher, Martin Pichler, Edgar Petru, Sigurd Lax, Johannes Haybaeck

**Affiliations:** 1 Institute of Pathology, Medical University Graz, Auenbruggerplatz 25, A-8036 Graz, Austria; E-Mails: elisabeth.smolle@stud.medunigraz.at (E.S.); valentin.taucher@stud.medunigraz.at (V.T.); 2 Department of Internal Medicine, Division of Clinical Oncology, Medical University Graz, A-8036 Graz, Austria; E-Mail: martin.pichler@medunigraz.at; 3 Department of Obstetrics and Gynecology, Medical University Graz, A-8036 Graz, Austria; E-Mail: edgar.petru@medunigraz.at; 4 Department of Pathology, General Hospital Graz West, Goestinger Straße 22, A-8020 Graz, Austria

**Keywords:** ovarian cancer, molecular carcinogenesis, targeted therapy

## Abstract

Ovarian carcinoma (OC) is the most lethal gynecological malignancy. Response to platinum-based chemotherapy is poor in some patients and, thus, current research is focusing on new therapy options. The various histological types of OC are characterized by distinctive molecular genetic alterations that are relevant for ovarian tumorigenesis. The understanding of these molecular pathways is essential for the development of novel therapeutic strategies.

**Purpose:**

We want to give an overview on the molecular genetic changes of the histopathological types of OC and their role as putative therapeutic targets.

**In Depth Review of Existing Data:**

In 2012, the vascular endothelial growth factor (VEGF) inhibitor, bevacizumab, was approved for OC treatment. Bevacizumab has shown promising results as single agent and in combination with conventional chemotherapy, but its target is not distinctive when analyzed before treatment. At present, mammalian target of rapamycin (mTOR) inhibitors, poly-ADP-ribose polymerase (PARP) inhibitors and components of the EGFR pathway are in the focus of clinical research. Interestingly, some phytochemical substances show good synergistic effects when used in combination with chemotherapy.

**Conclusion:**

Ongoing studies of targeted agents in conjunction with chemotherapy will show whether there are alternative options to bevacizumab available for OC patients. Novel targets which can be assessed before therapy to predict efficacy are needed. The assessment of therapeutic targets is continuously improved by molecular pathological analyses on tumor tissue. A careful selection of patients for personalized treatment will help to reduce putative side effects and toxicity.

## 1. Pathology and Biology of Ovarian Carcinoma

In the past, ovarian carcinoma (OC) has been considered as one single disease. However, ovarian carcinoma comprises a variety of tumors with various histopathological features and expresses different biological behavior. Currently, therapy rather depends on tumor stage and grade than on the histological type [1]. It can be expected that a more individual approach of treating ovarian carcinoma will be selected in the future since several phase III studies investigating targeted therapies are underway [1]. The search for novel therapeutic targets is a growing focus of research and crucial for the development of new drugs [2].

The World Health Organization (WHO) classifies ovarian neoplasms according to their histological differentiation, namely epithelial, sex cord-stromal and germ cell neoplasms [3]. Epithelial ovarian tumors represent the largest group and are basically subdivided into serous, mucinous, endometrioid, clear cell and transitional cell tumors; the latter including Brenner tumors [3]. Among these groups of tumors, three categories are distinguished according to the biological behavior: benign, borderline and malignant. Several rare malignant neoplasms complete the category of epithelial ovarian tumors, such as mixed carcinoma, carcinosarcoma and undifferentiated carcinoma.

Among the borderline tumor category, the serous type and the endocervical variant of the mucinous type may present at advanced stages II and III and are associated with recurrence in about 10% of cases. Whereas, the other histological types are typically stage I and show a benign course. Serous neoplasms are the most frequent category, encompassing more than 50% of ovarian tumors. Serous carcinomas are currently separated into two histologically and biologically distinctive subtypes, low grade and high grade, based on the degree of nuclear atypia and the amount of mitoses. Low grade serous carcinomas are infrequent and typically associated with serous borderline tumors [1]. Recent investigations indicate that the tube is probably the place where serous ovarian carcinomas originate [4–6]. Especially the tubal fimbria seems to play an important role in carcinogenesis. There is evidence that the mechanism of tumor formation in the tube is basically very different compared to that of the ovary [4,7,8]. Primary mucinous carcinomas of the ovary account for about 10% of epithelial ovarian neoplasms [9] and are most common of the so-called intestinal/enteric type. Mucinous carcinomas are often associated with a mucinous cystadenoma and/or a mucinous borderline tumor, and are most frequently unilateral and diagnosed at stage I [1]. Endometrioid and clear cell carcinomas are frequently associated with endometriosis, which is generally considered a risk factor of ovarian carcinoma. In particular, 2% of clear cell carcinoma patients, 9% of endometrioid carcinoma patients, and, interestingly, also 2% of patients with low grade serous carcinoma show a history of endometriosis [10]. Most endometrioid carcinomas are well or moderately differentiated, *i.e.*, low grade and only a small subset is poorly differentiated, *i.e.*, high grade. The distinction of some histological types may be difficult, in particular between high grade endometrioid and high grade serous carcinomas and between true clear cell carcinomas and other types of carcinomas featuring areas of clear cells [11].

Recently, the differential diagnosis of ovarian carcinomas has been supported by immunohistochemistry by a selection of antibodies drawn from the molecular tumorigenesis. In particular, a panel of estrogen (ER) and progesterone (PR) receptors, p53, PTEN and Wilms Tumor Gene Product 1 (WT1) may be helpful to type carcinomas with indefinite histological features. Only serous carcinomas are generally WT1 positive and high grade serous carcinomas typically show diffuse intense p53 immunoreactivity [11]. On the other hand, endometrioid and clear cell carcinomas may show a loss of PTEN, which is usually caused by mutation. Immunoreactivity for ER and PR is typical for endometrioid and serous carcinomas and less intense or absent in clear cell and mucinous carcinomas. p53 is typically weak or negative in clear cell carcinoma, but may be positive in a subset of clear cell and poorly differentiated emdometrioid carcinomas; yet, in contrast to high grade serous carcinomas p53 shows a heterogeneous pattern. Finally, clear cell carcinoma is characterized by a frequent expression of hepatocyte nuclear factor 1 (HNF1) which is not found in the other histological subtypes.

## 2. In Depth Review of Existing Data

### 2.1. Different Molecular Genetic Pathways and Putative Molecular Targets in Ovarian Cancer

**Figure 1:**
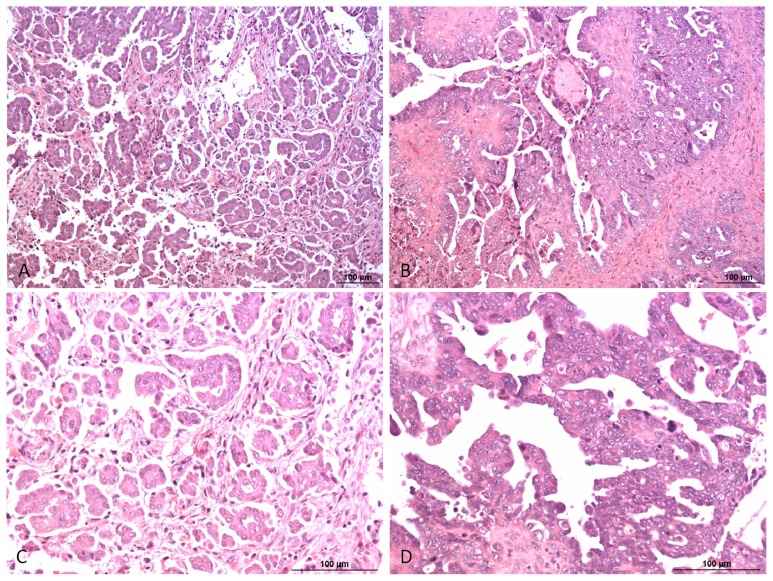
Type I/low grade (**A**, **C**) and type II/high grade (**B**, **D**) serous ovarian carcinoma. High grade serous carcinoma is characterized by a significantly higher degree of nuclear atypia and higher number of mitosis compared to low grade serous carcinoma. The papillae are less well preserved in high grade compared to low grade serous carcinomas. HE, 100× (**A**, **B**) and 200× (**C**, **D**).

Recent studies have led to a new model of explanation for OC carcinogenesis (Figure 1). This dualistic model divides epithelial OC into two categories: Type I comprises low-grade serous, low-grade endometrioid, clear cell and mucinous carcinomas and Brenner tumors. They mostly present at stage I (tumor is confined to the ovaries) and feature certain mutations, for example *K-Ras*, *B-Raf* or *PTEN*. The tumorigenic pathway in type I carcinomas is characterized by the development through atypically proliferating or borderline tumors which can be considered as an adenoma-carcinoma sequence. The residues of the benign and/or the borderline stages are frequently found in association with the carcinoma. Type I tumors develop slowly over a longer period of time, are not associated with dramatic clinical symptoms and usually are detected by chance during routine examination. They show a favorable prognosis even at higher stages. Progression into type II carcinomas seems to occur only in a small subset of type I carcinomas, in particular low grade serous and endometrioid carcinomas. Apart from mutations of *K-Ras*, *B-Raf* and *PTEN*, type I carcinomas also feature microsatellite instability in about 15%. *p53* mutations are rarely present in type I carcinomas but may occur during progression into type II carcinomas.

**Figure 2:**
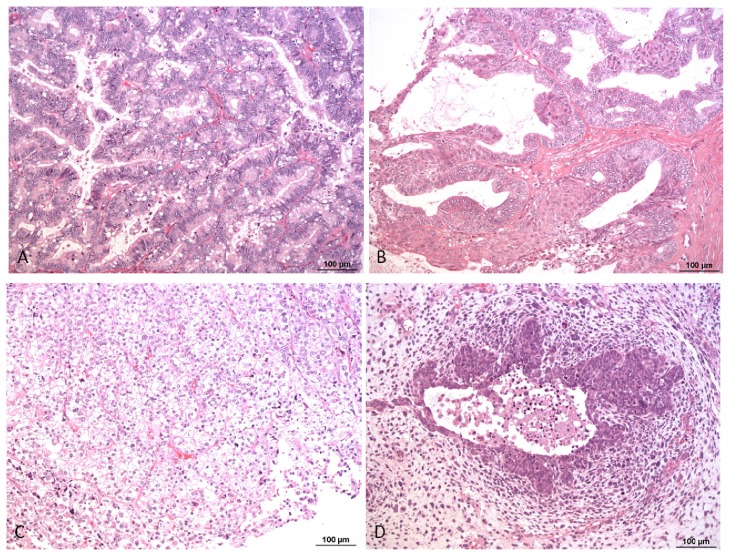
Mucinous (**A**), endometrioid (**B**), clear cell carcinoma (**C**) and mixed malignant mesodermal tumor (MMMT) (**D**). HE, 100×.

**Figure 3:**
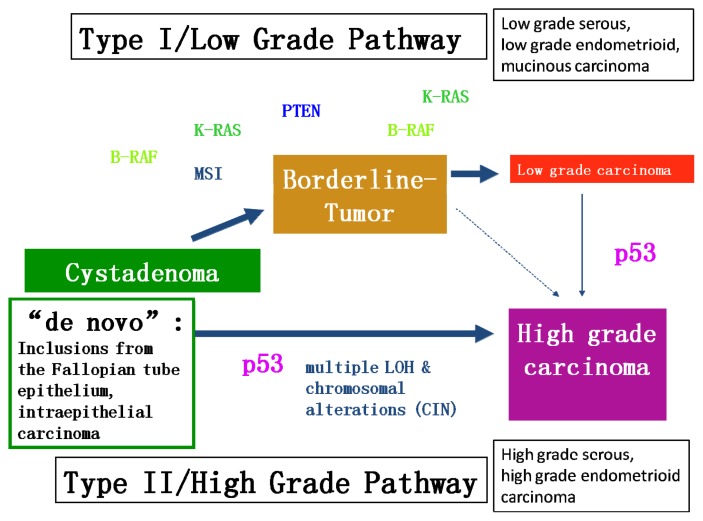
Molecular tumorigenesis of type I and type II ovarian carcinoma (modified according to Kurman, Shih 2004, Lax 2009).

High-grade serous, high-grade endometrioid and undifferentiated carcinomas, as well as malignant mixed mesodermal tumors count among type II (Figure 2). They are all histologically high grade neoplasms with aggressive course and unfavorable prognosis. Typically, they are not or only exceptionally associated with borderline tumors and, therefore, considered to develop without a well-defined precursor lesion “*de novo*”. Type I and type II tumors also differ in molecular tumorigenesis (Figure 3). Unlike type I, type II carcinomas often present at advanced stage and have a high frequency of *TP53* mutations, whereas mutations occurring in type I carcinomas are rarely found. Type II carcinomas also often feature alterations of the tumor suppressor genes breast cancer 1, early onset (*BRCA1*) and breast cancer 2, early onset (*BRCA2*) and are in general genetically unstable [12]. Also, the chromatin remodeling gene *Rsf-1* has been recently demonstrated in high grade ovarian serous carcinomas. Abundant *Rsf-1* expression can contribute to genomic instability, which favors tumor growth and has anti-apoptotic effects, which is typical for type II ovarian carcinomas [13].

Serous tumorigenesis has been a focus of research over the last two decades. Well defined precursor lesions were described for serous carcinomas of the endometrium and the Fallopian tube, named intraepithelial carcinoma (serous endometrial intraepithelial carcinoma (SEIC) and serous tubal intraepithelial carcinoma (STIC), respectively). These intraepithelial carcinomas are flat lesions consisting of highly atypical cells, which frequently harbor *p53* mutations. Neoplastic cells with mutated *p53* show either strong or flat negative immunoreactivity. In the past, it has been hypothesized that high grade ovarian serous carcinomas could develop from inclusion cysts by malignant transformation. Recently, a unifying model for ovarian and tubal neoplasms tried to synthesize the dualistic model of ovarian carcinoma with the role of the Fallopian tube in the development of serous carcinomas of the female genital tract [14]. According to this proposed model, most serous carcinomas develop from Fallopian tube epithelium that is implanted into the ovaries. High grade serous carcinomas either develop from tubal intraepithelial carcinoma (TIC), which typically occurs in the fimbria, or from malignant transformation of serous inclusion cysts in the ovary. In contrast, low grade serous carcinomas develop from serous inclusion cysts through borderline tumors. Endometrioid and clear cell carcinomas arise within endometriosis, which typically results from implantation of endometrial tissue into the ovaries. It has been further proposed that based on preliminary data mucinous and transitional (Brenner) tumors may arise from transitional-type epithelial nests at the tubal-mesothelial junction by a process of metaplasia [14]. Clear cell carcinomas are considered to develop in the background of endometriosis and frequently harbor mutations in the *ARID1A* gene [15].

Summing up these recent findings of carcinogenesis in the ovary, it is evident that OC is not a single disease but comprises a heterogeneous group of tumors that can be classified from their distinctive morphologic and molecular genetic features [14].

### 2.2. Putative Molecular Targets

#### 2.2.1. *BRCA1* and *BRCA2*

The *BRCA1* and *BRCA2* genes are located on chromosomes 17q21 and 13q12, respectively. Germ line mutations of these two genes are an important cause of hereditary breast cancer. The mutations are found at different sites of these genes and tremendously increase the risk of developing breast and ovarian cancer. In particular, in *BRCA1* mutation carriers, the cumulative life time risk for breast cancer is about 70%; for ovarian cancer 40%; and, in *BRCA2* mutation carriers, 50% for breast and 10% for ovarian cancer. It is currently estimated that about 30%–50% of hereditary breast and ovarian cancers develop in *BRCA1* and *2* mutation carriers. This accounts for about 5%–10% of overall breast and ovarian carcinoma incidence [16]. In addition, the overall risk of developing any sort of cancer also seems to be elevated by 20%–60%. In *BRCA1* and *2* germline mutation carriers, somatic inactivation of the remaining wild-type allele is required, which means that a somatic mutation has to be dominant [17,18].

*BRCA1* and *BRCA2* seem to be involved in the repair of DNA double strand breaks, and in the regulation of transcription [19,20]. In particular, *BRCA1* repairs DNA breaks by homologous recombination, which is a repair mechanism widely used by cells. It allows the exchange of nucleotide sequences between two similar or identical molecules of DNA. *BRCA1* moves to the locus of DNA break site, recruited there by the histone protein *H2AX* [21]. It was suggested, that *BRCA1* and *H2AX* function as initiators of DNA break repair by making the respective foci accessible to ligating proteins [21]. *BRCA1* also acts in the alternative non-homologous end-joining (NHEJ) pathway, while *BRCA2* only works in the repair process of double strand breaks using homologous recombination [22]. The NHEJ pathway is highly conserved by direct repair of DNA strand breaks. Ineffective NHEJ and ineffective repair of DNA strand breaks by homologous recombination can lead to translocations and other hallmarks of cancer [23]. Despite being a highly effective DNA repair mechanism, homologous recombination might have the harmful side effect of altering *BRCA1* and *2* gene function. It seems that cells which lack functioning *BRCA1* or *BRCA2* are more likely to accumulate chromosomal abnormalities such as aneuploidy or the amplification of the centrosome [24].

The somatic loss of the functional *BRCA* gene product was also observed in sporadic ovarian carcinomas. Loss of heterozygosity of the *BRCA1* gene was found in 50%–70% of sporadic ovarian carcinomas and loss of heterozygosity of *BRCA2* was found in 30%–50% [25,26]. This suggests that in the development of sporadic breast and ovarian carcinomas, the somatic loss of *BRCA* alleles might have an important pathophysiological role, similar to germline point mutations [27].

#### 2.2.2. *KRAS* and *BRAF* Mutations Lead to the Activation of the MAPK/ERK Pathway

Mutations in *KRAS* and *BRAF* typically occur in the so-called type I ovarian carcinomas; in particular in low grade serous, mucinous and endometrioid carcinomas [28,29]. *KRAS* and *BRAF* mutations lead to constitutive activation of the MAPK/ERK pathway [30,31]. *ERK* activates downstream targets in the nucleus and cytoplasm [32,33]. Hormones and growth factors that trigger signaling via mitogen-activated protein kinases (MAPKs) use two classes of surface receptors, receptor tyrosine kinases (*RTK*) and G protein coupled receptors (*GPCR*). MAPKs are involved in the transduction of the signals of several cytokines, growth factors and proto-oncogenes [31].

For ovarian carcinomas, important hormones using the ERK pathway via G-protein coupled receptors are Gonadotropins and Gonadotropin-releasing hormones. In the beginning of their signal transduction cascade, the extracellular mitogen binds to the membrane receptor, which allows *KRAS* (which yields GTPase function) to exchange its guanosine diphosphate (GDP) for guanosine triphosphate (GTP) and to change to its active form. Thus, it can activate *BRAF*, which activates *MEK* and consecutively *ERK* is activated. *ERK* can subsequently activate transcription factors, such as myc or elk-1.

The MAPK pathway has oncogenic potential, either through permanent signaling activity as a result of *KRAS* or *BRAF* mutation, or by involvement of induction of cell replication. Since activation or overexpression of upstream factors like *KRAS* or *BRAF* can lead to constitutive activation of ERK, this may in turn activate downstream protein kinases or transcription factors that are likely to enhance tumor development [32,33].

MAPKs also play a role in the stimulation of ovarian cancer cell growth by membrane receptor signals for Gonadotropins. Gonadotropins are a group of hormones to which belong the follicle stimulating hormone (FSH), the luteinizing hormone (LH) and human chorionic gonadotropin (hCG). FSH and LH receptors are often expressed on ovarian carcinoma cells, and may therefore contribute to signaling transduction by MAPK [34,35].

#### 2.2.3. *EGFR* and the Consecutive Activation of *AKT*

Epidermal growth factor receptor (*EGFR*) is expressed in 70% of ovarian carcinomas [36]. It can be activated by various ligands, such as EGF and TGF [37] and plays a role in enhancing and inhibiting tumor survival [38–40]. *EGFR* is also involved in tumor infiltration, metastasis and angiogenesis [41,42]. *AKT* is a major downstream factor of *EGFR* signaling [43]. Upon ligand binding to *EGFR*, *AKT* is activated by phosphorylation at Ser473 [43]. *AKT* is regularly overexpressed in OC and is associated with poor prognosis and aggressive tumor behavior [44,45]. Since the EGFR/AKT pathway is involved in various aspects of cancer proliferation like angiogenesis and metastasis, it is currently considered as an attractive target for therapeutic intervention.

#### 2.2.4. Integrin Inhibitors

Lately, research has been done on the application of integrin inhibitors as potential therapeutic agents in ovarian carcinoma. The initial step in ovarian carcinoma dissemination occurs by the attachment of carcinoma cells onto the peritoneal surface via integrins, and therefore targeting integrins seems a rational therapy approach. However, no integrin inhibitors have shown favorable effects so far [46].

#### 2.2.5. *GRP78* Expression

Another recent article proposes *GRP78* as a drug delivery system targeting ovarian carcinoma cells. *GRP78* upregulation is a cellular mechanism of response, caused by endoplasmic reticulum stress, which is commonly found in tumor cells. Since *GRP78* is abundantly found on ovarian carcinoma cell surfaces, the authors suggest the use of *GRP78* as a delivery system for cytotoxic substances [47].

#### 2.2.6. The p38alpha Pathway

The p38alpha pathway has recently been the focus of cancer research. Small compound inhibitors of p38alpha have already been evaluated in clinical trials showing promising results and may present a future therapeutic option for ovarian carcinoma. [48].

## 3. Diagnosis of Ovarian Cancer: Biomarkers and Imaging Techniques

For the diagnosis of ovarian tumors Doppler ultrasound, MRT and computed tomography as well as the assessment of biomarkers may be used. Early detection of OC is necessary to improve overall-survival, since only 25% of ovarian cancers are detected at stage I [49]. Among the serum markers, CA-125 receives the most attention, but sensitivity and specificity are not high enough for its role as a single screening test. Its sensitivity can be enhanced by using a panel of biomarkers. In addition, monitoring of biomarkers over time may be of value [49]. At present, the combination of transvaginal sonography and the evaluation of biomarkers are standard for OC diagnosis. An algorithm has been developed based on serial CA-125 values which refers patients at high risk for OC to transvaginal sonography [50].

Nearly 2% of adnexal masses turn out to be carcinomas or borderline tumors [51]. According to Marret, every suspicious ovarian mass would need expert sonography. Transvaginal sonography has considerable advantages compared to conventional transabdominal sonography. It is important to look for papillary formations inside ovarian cysts and for non-hyperechoic solid components, since these features are strong predictors of malignancy [51]. The evaluation of tumor vascularity by Doppler energy is especially useful to distinguish benign from malignant lesions [51]. Conventional ultrasound supplemented with three-dimensional (3D) ultrasound and three-dimensional power-Doppler (3DPD) ultrasound seems to be useful for the preoperative distinction of ovarian lesions [52].

CA-125 is the most valuable tumor marker for ovarian cancer. According to a study in which patients with ovarian metastases were compared to patients with primary OC, a CA-125 level >170 U/mL predicted primary ovarian cancer in >95% of the patients. In this study, CT imaging was also performed. In patients with primary OC, the CT scan rather than ultrasound showed omental involvement of the ovarian tumor mass and ascites, in comparison to ovarian metastases [53].

Additionally, it seems necessary to evaluate biomarkers to achieve higher levels of diagnostic sensitivity and specificity. Currently, more than 30 biomarkers are tested alone and also in combination with CA-125, e.g., mesothelin, osteopontin or kallikrein [50]. Mass spectroscopy of a patient’s serum is a novel approach to diagnosis. A specific pattern of peaks in the mass spectroscopy has been found, that is predictive for OC [50]. Relevant cancer markers are now determined by several study groups and put on platforms to permit the simultaneous assessment of a panel of markers using only small volumes of serum [50]. It has been demonstrated that the diagnostic value to predict malignancy is higher when a combination of biomarkers, as e.g., Human Epididymal Protein 4 (HE4), CA-125 and carcino-embryonic antigen (CEA) are determined. These factors also have to take into account the patient’s age, compared to the determination of CA-125 alone [54].

Recent data indicate that the folate-receptor 1 (FOLR1) is significantly elevated in the serum of OC patients compared to healthy controls and patients with benign gynecological tumors. FOLR1 strongly correlated with CA-125 and may be a potential candidate to serve as a biomarker for OC [55].

Recently, a method has been developed to quantify cancer biomarkers in biological fluids using small optical microresonators that support whispering gallery mode resonance. These optical microresonators are emerging as a new promising and powerful method for the detection of biomarkers in complex biological fluids by biosensing. Huckabay *et al*. report a method which allows detecting the OC marker CA-125 in buffer via whispering gallery mode imaging using a technique based on fluorescence imaging. In addition, the OC markers osteopontin and prolactin have also been investigated [56].

There is also a new optical imaging technique, partial wave spectroscopic microscopy that can assess the nanoscale macromolecular fluctuations of density within cells using a biomarker. The authors, who developed this novel technique, investigated endometrial and endocervical columnar cells referring to the concept of field carcinogenesis. This evaluation shows that there is a significant increase of the investigated biomarkers in columnar epithelial cells of OC patients compared to the controls [57].

Summing up, diagnosis of OC shall be made using imaging techniques, first of all transvaginal sonography, plus measuring a panel of biomarkers [49].

## 4. Treatment Modalities

Currently, therapy of ovarian carcinoma is based on maximum primary surgical debulking and adjuvant chemotherapy including platinum and a taxane [58,59]. However, the recurrence rate is high despite of adequate primary treatment [60]. This is mainly due to late diagnosis since 70% of the cases are diagnosed at advanced stage (FIGO Stages IIB–IV) [61]. Nevertheless, only 10%–15% of ovarian carcinoma patients achieve long-term remission and overall five-year survival is lower than 25% [62–65]. In late stage disease, five-year survival is less than 40%. In contrast, five year survival is considerably higher in stage I–II disease with more than 80% [61].

Unfavorable prognosis is in part caused by the early development of chemotherapy resistance [66]. Even though initial response is as high as 70% to 80%, most patients ultimately die of recurrence [64,65]. Treatment of clear cell carcinomas by chemotherapy is particularly problematic as they may be chemoresistant [67,68].

Second-line treatment is needed in the majority of patients. Platinum sensitivity is a good predictor of response. Platinum refractory disease” and “platinum-resistant” disease has a bad prognosis while patients with a long interval between diagnosis and recurrence show a better survival. This disease is defined as “platinum sensitive”.

For recurrent ovarian carcinoma patients with partial platinum sensitivity, *i.e.*, recurrence between 6 and 12 months after the end of platinum-based therapy, trabectedin, a marine-derived anticancer agent, has shown preferential activity [69]. It acts via binding to a DNA minor-groove [70].

The monoclonal antibody bevacizumab, a VEGF inhibitor, is approved for ovarian cancer in the first line and in platinum-sensitive recurrence [60,71,72]. Bevacizumab causes hypertension in a significant proportion of patients. The greatest effect of bevacizumab was seen in patients with a high risk for progression, *i.e.*, extensive disease and significant residual tumors. Bevacizumab can decrease ascites in ovarian carcinoma [73].

The mammalian target of rapamycin (mTOR) is responsible for cell growth and proliferation, interacting with VEGF and platelet derived growth factor (PDGF); the latter results in activated angiogenesis [74]. In clear cell ovarian carcinoma mTOR inhibitors have single-agent activity [75]. MTOR-inhibitors might be particularly effective in combination with bevacizumab since synergistic effects have been detected [76–78].

Poly-ADP-ribose polymerase inhibitors (PARP inhibitors) belong to a family of multifunctional enzymes with promising effects in ovarian carcinomas featuring *BRCA1* or *2* mutations. These drugs block base excision repair and lead to the accumulation of DNA single-strand breaks. The latter subsequently cause DNA double-strand breaks at replication forks [69]. In normal cells these double-strand breaks are repaired in the presence of the tumor suppressor proteins *BRCA1* and *2* [69]. In the absence of these proteins the lesions cannot be repaired, resulting in cell death. Thus, PARP inhibitors are suitable for the treatment of tumors with dysfunctional DNA repair. Three phase II studies with the PARP inhibitors olaparib and iniparib have demonstrated activity in platinum-sensitive ovarian carcinoma [78–81]. A preliminary study has demonstrated that ovarian carcinoma patients with *BRCA1* or *2* mutations respond better to olaparib than those without mutations [80]. Olaparib seems to be associated with improved progression-free survival after conventional chemotherapy [79] and therapeutic response in both platinum-resistant and platinum-refractory disease [82].

Research is also focusing on epidermal growth factor receptor (EGFR) dependent pathways [58]. The EGFR receptor is overexpressed in 30%–98% of ovarian carcinoma [83]. The EGFR antibody cetuximab and the EGFR tyrosine kinase inhibitors lapatinib and erlotinib have not shown clinically significant activity in ovarian carcinoma yet [84,85] but may cause severe toxic and hematologic side effects [86,87].

Reports on Her2 expression in OC show divergent results [74]. Both overexpression and amplification are more prevalent in high-grade serous carcinomas, whereas low-grade serous and endometrioid carcinomas usually do not overexpress Her2 [88]. A few studies have shown moderate activity of anti-Her2 therapy with trastuzumab and pertuzumab [89,90]. Anti-Her2 therapy has shown particular activity in patients with Her2 overexpression in preliminary studies [90].

Farletuzumab is a humanized, IgG monoclonal antibody with high affinity for folate receptor alpha, a 38 kDa protein that is overexpressed in about 90% of OC [91]. The degree of folate receptor alpha expression correlates with tumor stage and grade [92]. In normal tissue, folate receptor alpha is largely absent, making it a relevant and attractive therapeutic target [91,93]. Farletuzumab has shown good antitumoral activity in preclinical xenograft models and has shown promising results in early phase trials [91,92]. A phase 1 dose escalation study has shown no dose-limiting toxic side effects or severe adverse effects [92]. A phase 2 efficacy and safety study using a combination of farletuzumab with carboplatin and taxane in patients with platinum-sensitive OC showed improved response rates and a longer time to progression [92]. The combination of farletuzumab, carboplatin and Pegylated Liposomal Doxorubicine (PLD) has a good safety profile, according to a study with platinum-sensitive OC patients following first or second relapse [92].

Malignant ascites affects about 10% of patients suffering from recurrent OC [94]. The concomitant symptoms include abdominal pressure, dyspnea, bloating, pelvic pain and bowel or bladder dysfunction. Treatment options for malignant ascites in OC patients include the use of antiangiogenic agents, namely bevacizumab and vascular endothelial growth factor inhibitors and also nonangiogenic drugs such as catumaxomab [94]. Catumaxomab is a rat/murine hybrid bispecific (anti-human epithelial cell adhesion molecule [EpCAM] and anti-CD3) monoclonal antibody [95]. Treatment of malignant ascites with paracentesis alone is much less effective than paracentesis followed by intraperitoneal catumaxomab treatment. Paracentesis-free survival was significantly longer, according to a phase II/III trial with patients suffering from recurrent, symptomatic malignant ascites [95]. Additionally, catumaxomab treatment was associated with a reduction of ascites signs and symptoms and with delayed deterioration regarding health-related quality of life. Generally, catumaxomab is well tolerated. The most frequent adverse effects include cytokine-release-related symptoms, but these were mostly mild and manageable with standard antipyretics [95].

Epigenetic changes in cells, such as hypoacetylation of histones and abnormal DNA methylation, may also promote tumorigenesis and lead to chemotherapy resistance. A phase II study with decitabine, an agent leading to DNA hypomethylation, has shown effectivity in platinum-resistant disease [78,96]. Currently, the histone deacetylase inhibitor belinostat and the proteosome inhibitor carfilzomib are evaluated [73].

The MAPK/ERK pathway can contribute to therapy induced tumor-growth suppression. In particular, the synthetic retinoid CD437 seems to be capable of inhibiting growth and inducing apoptosis in the ovarian cancer cell line CA-OV-3. In addition, p38, which influences growth inhibition, seems to be induced independently [97]. The response of cancer cells to the chemotherapeutic drug cisplatin is dependent on MAPKs, by induction or suppression of apoptosis [98].

The *MAPKs* and *BRAF* are involved in cellular growth regulation and can trigger carcinogenesis [73]. In low-grade ovarian carcinoma, BRAF mutations are frequently found and lead to activation of the MAPK-pathway [73]. Thus, the BRAF/MAPK pathway is a potential therapeutic target. BRAF inhibitors such as vemurafenib and dabrafenib and the MEK inhibitor trametinib have shown significant activity in BRAF-mutated melanoma. The MET tyrosine kinase cell surface receptor is linked to this pathway and its inhibition leads to the suppression of RAF and MAP kinase activity. Recently, the MET inhibitor cabozantinib has shown efficacy in ovarian carcinoma independently from platinum sensitivity [78].

Recently, some anti-inflammatory phytochemicals have been tested, which may change the immunosuppressive microenvironment [73]. Such phytochemicals are mainly natural colorants extracted from fruits or vegetables. They have an inhibitory effect on the arachidonic acid pathway and therefore act similarly to non-steroidal anti-rheumatics [99]. Phytochemicals include the following flavonoids: apigenin, baicalein, genistein, luteolin, quercetin, wogonin curcumin, and the antioxidants epigallocatechingallate and oridonin. They repress NF-kappaB, a proinflammatory transcription factor and inhibit proinflammatory cytokines [73,100–102]. NF-κB has a paradoxic role in carcinogenesis. On one hand activated NF-κB promotes apoptosis, but on the other hand, inhibition of NF-κB may lead to pro-apoptotic effects and inhibits chemotherapy-resistant ovarian carcinoma cell growth [103]. Anti-inflammatory phytochemicals also stabilize *p53*, a tumor suppressor gene [73,104]. These effects may result in the prevention or delay of resistance to chemotherapy [73]. Apigenin, genistein, kaempferol, luteolin, and quercetin have been demonstrated to inhibit VEGF *in vitro* [73,105]. Oridonin and wogonin may also contribute to the suppression of cancer stem cells by down-regulating the surface marker EpCAM [73].

The role of non-steroidal anti-inflammatory drugs (NSAIDs) in ovarian carcinoma is incompletely understood. There is epidemiological evidence for an association of increased NSAID/aspirin intake and the prevention of colon cancer [73,106]. A meta-analysis and one prospective cohort study on the association of NSAID intake and ovarian carcinoma risk did not find a risk reduction [73,107]. In another trial, the beneficial effect of NSAIDs in the prevention of ovarian carcinogenesis was demonstrated [73,108].

A potential next-generation therapy for ovarian carcinoma is the use of microRNA therapeutics [73]. In mouse models, let-7 has been demonstrated to repress cell proliferation in breast cancer cells [109,110]. However, at present, their mechanisms of action are poorly understood [73].

## 5. Discussion and Conclusion

Ongoing studies of targeted agents in conjunction with chemotherapy will reveal whether other options than bevacizumab will be effective in ovarian carcinoma. Since these agents are expensive and may also cause significant toxicity, novel specific biomarkers are urgently needed to predict their efficacy and to justify their use. Mouse models are good candidates to test new approaches to ovarian carcinoma therapy.

The fact that different histological types of ovarian carcinoma feature specific signaling characteristics, may be used to target molecular objectives and to provide individualized therapy options [111]. There is growing evidence that epithelial ovarian cancer is a heterogeneous disease that needs a tailored approach based on the underlying molecular genetic changes. Several drugs targeting components of the phosphoinositide 3-kinase/protein kinase B (PKB)/Akt (PI3K/Akt) pathway have already been designed and some have also been tested in clinical trials [111]. However, since there is minimal experience with these drugs and only a few patients are willing to take the risk of testing, mouse models have been established to gain more preclinical information. Wu *et al*. have developed a mouse model of endometrioid adenocarcinoma by inactivating the tumor suppressor genes *PTEN* and Adenomatous polyposis coli (*APC*) in the murine ovarian epithelium [59]. For this purpose, the “Cre/lox” system was used. In the ovarian carcinoma mouse model, the mice had *loxP* sites in the introns of the *PTEN* and *APC* genes. A virus expressing the Cre recombinase was injected into the murine ovarian bursa. Subsequently, the mice developed tumors that were similar to human ovarian endometrioid tumors. In these mice, the neoplasms rapidly progressed and all mice died within 19 weeks after Cre had been delivered to the ovarian epithelium. Treatment with Rapamycin, an inhibitor of mTOR (a downstream effector of AKT) distinctly showed growth inhibition of the cancers in the mouse model [111]. The effects on tumor growth were investigated with non-invasive methods taking advantage of bioluminescence imaging. In the future, such mouse models are expected to provide further insight into the biology of OC and to reveal new putative points of action for targeted treatment.

Further development of our understanding of ovarian cancer pathogenesis and a deeper knowledge of the underlying molecular alterations will be crucial for a successful implementation of targeted therapy of ovarian carcinoma. It will be the pathologists’ role to analyze tumor tissue for key molecular targets in ovarian carcinoma, as is today routinely performed for targeted therapy of colorectal and non-small cell lung carcinoma and melanoma.
